# Early peripheral blood gene expression predicts 90-day outcomes following subarachnoid hemorrhage

**DOI:** 10.1186/s12974-025-03630-0

**Published:** 2025-12-07

**Authors:** Bodie Knepp, Garreck H. Lenz, Frank R. Sharp, Fernando Rodriguez, Huimahn Alex Choi, Aaron M. Gusdon, Glen Jickling, Lara L. Zimmermann, Ryan Martin, Jeffrey Vitt, Ben Waldau, Brandon J. Cord, Alan Yee, Kwan Ng, Nerissa U. Ko, Heather Hull, Bradley P. Ander, Boryana Stamova

**Affiliations:** 1 Department of Neurology, School of Medicine, University of California at Davis, Sacramento, CA USA; 2 Department of Neurosurgery, UT Health Houston McGovern Medical School, Houston, TX USA; 3 Division of Neurology in Department of Medicine, University of Alberta, Alberta, Canada; 4 Department of Neurosurgery, School of Medicine, University of California at Davis, Sacramento, CA USA; 5 Department of Neurology, University of California at San Francisco, San Francisco, CA USA

**Keywords:** Subarachnoid hemorrhage, Outcome, Human, RNA-Seq, Genes, Machine-learning, Neutrophils, Biomarkers

## Abstract

**Background:**

Previous clinical, radiological and machine learning studies have predicted 90-day outcomes following subarachnoid hemorrhage (SAH). The present study was designed to determine whether early changes in mRNA expression of immune, clotting and other genes expressed in peripheral blood can predict patient outcomes at 90 days after SAH and possibly provide insights into the molecular factors that promote good versus poor outcomes.

**Methods:**

Peripheral blood was drawn after SAH and from vascular risk factor controls (VRFC) and RNAseq performed to measure mRNA expression. A mixed effects regression model identified potential predictors and machine learning algorithms derived the best predictors of 90-day SAH outcome as measured by modified Rankin Score (mRS) for a derivation cohort (23 Poor and 37 Good SAH Outcome patients, 48 VRFC). The model trained on the derivation cohort was then used to predict 90-day SAH outcome in an independent validation cohort (15 Poor and 23 Good SAH Outcome). Enrichment analyses for cell-type specific genes, canonical pathways, and biological processes were performed for the predictor genes.

**Results:**

The mixed effects regression on the derivation cohort yielded 94 genes from which 20 were selected through feature reduction. Machine learning algorithms were optimized to generate a model that predicted SAH 90-day outcome with AUC = 0.85, sensitivity = 87%, and specificity = 84% on cross-validation. Application of this model to the independent validation cohort yielded AUC = 0.84, sensitivity = 93%, and specificity = 74%. The 20 predictors were significantly enriched in genes from neutrophils and erythroblasts and in nine pathways including the Unfolded Protein Response, Neutrophil Degranulation, and Neutrophil Extracellular Trap Signaling.

**Conclusions:**

This discovery study demonstrates that a small panel of 20 genes expressed in peripheral blood after SAH has the potential for predicting 90-day outcomes following SAH. It also shows that neutrophils may be important drivers of SAH outcomes and could represent therapeutic targets.

**Supplementary Information:**

The online version contains supplementary material available at 10.1186/s12974-025-03630-0.

## Background

Subarachnoid hemorrhage (SAH) resulting from ruptured aneurysms and other causes represents approximately 5% of all strokes and is associated with high patient morbidity and mortality [1, 2]. Factors associated with neurological outcome following SAH include but are not limited to: age, admission neurological Hunt & Hess grade, aneurysm characteristics (such as location and size), SAH imaging severity, concurrent intraventricular or intracerebral hemorrhage (ICH), early rebleeding, delayed cerebral ischemia, vasospasm, time to surgical intervention and acute hydrocephalus [1, 3]. Approaches targeting some of these factors have decreased the 90-day mortality rate over time [4]. 

Predicting SAH outcomes is important because it might help guide treatment and allocation of resources, provide prognostic information for caregivers and patients, and could be used to help stratify patients for SAH clinical trials. A number of studies have utilized clinical scales to predict SAH outcomes, including the Glasgow Coma Scale [5], and a model consisting of Hunt & Hess Grade, Acute Physiology and Chronic Health Evaluation II physiologic score, age, and aneurysmal rebleed within 48 h [6]. Other models consisting of age, hypertension, and World Federation of Neurosurgical Societies (WFNS) scale with or without a neuroimaging model (such as Fischer grade, size and site of the aneurysm) have also been examined [7]. Additional studies have shown the combination of WFNS and Fischer grade were predictive of functional outcomes [8] as well as a combination of hemorrhage status (consisting of SAH thickness via Barrow Neurological Score and rebleeding events), age, treatment, WFNS, and hydrocephalus [9]. Radiological scores can also predict SAH outcome, with the Hijdra sum attaining the highest AUC (0.73) on cross-validation [10]. Artificial intelligence approaches have been shown to have similar performance to existing predictors of SAH outcome with fewer training cases required [11]. Limitations of clinical scales and radiographic scores exist [5] which highlight the need for biological biomarkers of SAH outcomes.

Serum and cerebrospinal fluid (CSF) biomarkers such as enolase, S100B, and glial fibrillary acidic protein (GFAP) in blood and cerebrospinal fluid (CSF) have been shown to predict SAH mortality [12]. Changes in brain tau [13] and CSF arginine/ornithine ratios are also associated with SAH outcome [14]. MicroRNAs have also been examined as predictors of aneurysmal SAH, its complications, and its prognosis [2]. With this background, we reasoned that immune and clotting pathways in peripheral blood might influence SAH outcomes and could be utilized for predicting outcomes. To address this possibility, we performed RNAseq on RNA from whole peripheral blood drawn at early times after SAH. A gene expression signature that was predictive of good or poor patient outcome was then produced using machine learning via a support vector machine (SVM) algorithm in a derivation cohort. The same gene expression signature was applied to a separate validation cohort to assess performance of predicting 90-day SAH patient outcomes.

## Methods

### Study participants and sequencing

A total of 148 subjects were enrolled in this prospective study in research hospitals in the United States and Canada between 2017 and 2023. The study protocol was approved by the Institutional Review Board (IRB) at the University of California, Davis with reliance agreements at the United States-based partnering sites at University of Texas Health, Houston and University of California, San Francisco. Separate IRB approval was obtained at the Canadian site of the University of Alberta in Canada. Written informed consent was obtained from all participants or their proxies.

Subarachnoid hemorrhage patients (SAH; *n* = 98) had subarachnoid blood identified by CT scan or lumbar puncture. This was followed by angiography and, if an aneurysm was identified, coiling or clipping was usually performed within 48 h of the ictus as indicated. After consent, whole blood was drawn into PAXgene tubes on average 2.5 days post *ictus* and always after surgery prior to any secondary event such as delayed cerebral ischemia (DCI). The 90-day outcomes were assessed over telephone between 90 and 97 days post *ictus* using the modified Rankin Scale (mRS). Outcomes were dichotomized, with good SAH outcome (*n* = 60) defined as mRS of 0–2 and poor SAH outcome (*n* = 38) defined as mRS of 3–6. For this study, DCI was defined as patients showing infarction assessed by MRI or CT between 3 and 14 d after the initial SAH after exclusion of procedure-related infarctions, and vasospasm was defined as angiographic narrowing due to vasoconstriction caused by subarachnoid blood.

Vascular risk factor control participants (VRFC) (*n* = 48) had at least one cardiovascular risk factor (hypertension, diabetes mellitus, hyperlipidemia). After consent, VRFC participants had whole blood drawn into PAXgene tubes. These participants had no history of any cerebrovascular disease and were matched for age, sex, and vascular risk factors to the patients in the derivation SAH cohort. Differences in demographic data between SAH and VRFC groups were analyzed using a two-tailed *t*-test and χ2 analysis where appropriate with *p* < 0.05 considered significant.

PAXgene tubes that stabilize the RNA in blood were stored frozen, and RNA was isolated and processed as previously described [15]. Libraries were prepared using the CORALL RNA-Seq V2 protocol including initial poly(A) selection, depletion of globin and breakthrough rRNA transcripts (with RiboCop + HMR treatment), and incorporation of unique molecular identifiers (UMI) and unique dual indexes (UDI) (Lexogen, Inc., Greenland, NH). High throughput sequencing (2 × 100 bp) was performed using a NovaSeq 6000 S4 (Illumina). For post-processing, raw reads were assessed for quality and composition using FastQC and aggregate data viewed with the MultiQC tool. Reads were passed through fastp for quality and adapter trimming, quality checked and then aligned to the HG38 human genome using STAR v2.7.11a. UMI-collapsed reads were aggregated to gene level counts tables for analyses.

### Data processing and filtering

Raw counts were CPM normalized, log_2_-transformed (offset 1xE-05), and separately quantile normalized for each of the derivation and validation cohorts. All filtering was conducted on the derivation cohort only. To only filter out genes with low expression in all groups, genes were removed if they had normalized expression values < 1.5 in 70% of subjects in all three of the groups (poor SAH outcome, good SAH outcome, VRFC). This filter retained any genes that were expressed in only one group as they may make good predictors. Genes with |coefficient of variation| > 50 within any group were removed to retain only genes with consistent expression within all groups. This resulted in a dataset of 12,338 candidate genes for further analysis.

### Identification of potential predictors in the derivation cohort

A diagram of the data analysis pipeline for the derivation and validation cohorts is shown in Supplementary Fig. 1. To identify potential predictors of SAH outcome, we used Partek Genomics Suite (PGS; Partek, St. Louis, MO) to run a mixed effects regression with group (Poor SAH Outcome, Good SAH Outcome, VRFC), age, sex, and study site (as a random factor). Potential predictors were defined as genes associated with both (1) group term as a whole (Benjamini-Hochberg adjusted (BH) *p* < 0.05) and (2) Poor vs. Good (BH *p* < 0.2, |fold change (FC)|>1.2). These genes were then used as potential predictors for feature reduction and model training. These analyses were performed on the derivation cohort which included 23 Poor SAH Outcome, 37 Good SAH Outcome, and 48 VRFC. The VRFC samples were only used in analysis of the derivation cohort to improve selection of the predictors and not in the training of the classification model or in the validation cohort for testing performance of the model.

### Training and validating the model

Feature reduction and model training were performed on only the derivation cohort of Poor and Good SAH Outcome participants in order to find the least number of genes that accurately predict SAH outcome with a classification model. We examined support vector machine (SVM), K nearest neighbors, nearest centroid, discriminant analysis, partial least squares, diagonal discriminant analysis, random forest, and logistic regression classification methods to identify which best predicted SAH Outcome. SVM models had the best and most consistent performance, and as such we focused on identifying the best possible combination of predictors and SVM parameters to create the most accurate model possible. A shrinking centroids filter was applied to the input set of predictors to identify the top 30 and followed by an exhaustive wrapper to identify the final 20 predictors. Different SVM models were created with varying configurations (linear, polynomial, radial, and sigmoid kernels with varying cost, gamma, and coef0 parameters) to determine the model to deploy on the validation cohort. Ultimately, the model was selected based on its overall performance (quantified by area under the curve, AUC) as well as the balance between sensitivity and specificity. We emphasized sensitivity in predicting the Poor SAH Outcome class as it may be more clinically relevant to identify all patients who are at risk of long-term severe morbidity who represent the greatest need for intervention. The final model was deployed on the validation cohort (15 Poor SAH Outcome, 23 Good SAH Outcome) to better estimate real-world performance. Additionally, we performed other feature reduction techniques and took the overlap between these 20 genes and those potential predictors to determine if we could identify fewer genes that still accurately predicted 90-day SAH outcome. Though we were able to achieve similar performance on the derivation cohort with these new models, the generalizability of these models on the validation cohort was inferior to our described 20-gene panel (data not shown).

### Biological interpretation of predictors

To examine the biological relevance of the final panel of predictors, we searched for enrichment in cell-type specific genes, canonical pathways, and biological processes. The predictors were analyzed for enrichment in blood cell type specific genes identified from the literature [16, 17] using a hypergeometric probability analysis (R function *phyper*, *p* < 0.05) in addition to gene lists from the literature associated with other neurodegenerative diseases [18–20]. We performed Ingenuity Pathway Analysis (IPA^®^, QIAGEN) core analyses on the predictors as previously described [21] in order to identify associated canonical pathways and biofunction terms (BH *p* < 0.05).

## Results

### Subject demographics

A total of 38 Poor SAH Outcome (mRS = 3–6 at 90 days post *ictus*), 60 Good SAH Outcome (mRS = 0–2 at 90 days post *ictus*), and 48 VRFC subjects were analyzed for this study. These subjects were then split into a derivation cohort (23 Poor SAH Outcome, 37 Good SAH Outcome, all 48 VRFC; demographics described in Table 1) and a validation cohort (15 Poor SAH Outcome, 23 Good SAH Outcome). Derivation Cohort SAH patients included 49 saccular aneurysms, 7 dissecting or blister aneurysms, 1 mycotic aneurysm, 2 aneurysms with unknown etiology, and 1 perimesencephalic SAH without an aneurysm. More causes of SAH were represented in the validation cohort to determine how generalizable the prediction profile was, including 23 saccular aneurysms, 1 dissecting or blister aneurysm, 2 aneurysms with unknown or other etiology, 9 perimesencephalic SAH without aneurysms, 1 SAH trauma patient without aneurysm, and 2 with Reversible Cerebral Vasospasm (RCVS) suspected to be related to undetected SAH. In the derivation cohort, all three groups were well matched for parameters listed in Table 1 except that hypertension was more prevalent in Poor SAH Outcome compared to Good SAH Outcome (*p* = 0.04). For SAH specific measures, groups were matched for DCI status and time post *ictus*, though vasospasm was more prevalent and Hunt & Hess scores were more severe at blood draw in the Poor SAH Outcome group (*p* = 0.02 and *p* = 0.048, respectively). Derivation cohort SAH participants were enrolled at an average of 51 h post *ictus* for Poor SAH Outcome and 64 h post *ictus* for Good SAH Outcome. On the 57 Derivation cohort SAH subjects with aneurysms of known etiology, Poor and Good outcome groups were well matched for saccular vs. non-saccular SAH. Matching was not performed in the validation cohort as unmatched groups are likely to give a better estimation of generalizability.

**Table 1:** Patient demographics

	Poor SAH Outcome	Good SAH Outcome	Vascular Risk Factor Controls	Poor vs. Good Statistics	Poor vs. VRFC Statistics	Good vs. Control Statistics
Subjects (#)	23	37	48	*NS*	*NS*	*NS*
Sex (#F, #M)	15, 8	28, 9	31, 17	*NS*	*NS*	*NS*
Hypertension (#)	16	15	23	*p* = 0.04	*NS*	*NS*
Diabetes (#)	3	1	6	*NS*	*NS*	*NS*
Hypercholesterolemia (#)	2	3	9	*NS*	*NS*	*NS*
Delayed Cerebral Ischemia (#)	6	4	-	*NS*	*-*	*-*
Vasospasm (#)	18	17	-	*p* = 0.02	*-*	*-*
Age, years (avg ± SD)	54.70 ± 12.83	56.54 ± 13.39	56.19 ± 15.86	*NS*	*NS*	*NS*
Time, hours (avg ± SD)	51.42 ± 57.85	64.00 ± 58.93	-	*NS*	*-*	*-*
Hunt Hess Score (avg ± SD)	3.26 ± 1.19	2.61 ± 1.09	-	*p* = 0.048	*-*	*-*
Interventional Management*	-	-	-	*-*	*-*	*-*
Coiling	12/23	19/36	8/45	*NS*	*-*	*-*
Clipping	7/23	13/36	6/45	*NS*	*-*	*-*
Lumbar Drain	2/22	1/35	0/45	*NS*	*-*	*-*
External Ventricular Drain	21/22	29/37	1/45	*NS*	*-*	*-*

Demographics of the 23 Poor SAH Outcome, 37 Good SAH Outcome, and 48 control participants. On the right, statistical significance between groups for the listed parameters

*NS* - Non-significant (*p* ≥ 0.05)

*for interventional management, data is presented as number of patients/number of patients with data available. Interventional management is presented for Vascular Risk Factor Controls as some controls had unruptured aneurysms; however, statistical comparisons of SAH versus control was not conducted as it is not expected control patients would undergo the same interventions as SAH patients

### Potential predictor identification, model training, and cross-validation performance on derivation cohort

A total of 94 genes passed both criteria (group BH *p* < 0.05; Poor vs Good SAH Outcome, BH *p* < 0.2, |FC|>1.2) and were selected as potential predictors for input to feature reduction. The 94 genes were then reduced to the best 20 that represent the final predictive signature (Table 2). Of the various predictive models, we selected a model which correctly predicted 20 of 23 Poor SAH Outcome and 31 of 37 Good SAH Outcome subjects in a 10-fold cross validation (polynomial kernel, gamma: 10^− 5^, coef0: 0, degree: 3). The model attained an AUC of 0.85, a sensitivity of 87%, and a specificity of 84% (Fig. 2B). Though other models attained a higher overall AUC (highest AUC of 0.87), this one was selected because it had a better balance of sensitivity and specificity, and it performed better at predicting Poor than Good SAH Outcome patients.

**Table 2:** 20 predictors of poor vs. good 90 day SAH outcome

Ensembl ID	HGNC Gene Symbol	Gene Name	Molecule Type	Fold Change (Poor vs. Good SAH Outcome)
ENSG00000101425	BPI	bactericidal permeability increasing protein	transporter	2.38
ENSG00000092067	CEBPE	CCAAT enhancer binding protein epsilon	transcription regulator	1.53
ENSG00000102265	TIMP1	TIMP metallopeptidase inhibitor 1	cytokine	1.48
ENSG00000100300	TSPO	translocator protein	transmembrane receptor	1.44
ENSG00000021355	SERPINB1	serpin family B member 1	other	1.38
ENSG00000085117	CD82	CD82 molecule	other	1.36
ENSG00000106330	MOSPD3	motile sperm domain containing 3	other	1.36
ENSG00000104447	TRPS1	transcriptional repressor GATA binding 1	transcription regulator	1.35
ENSG00000065989	PDE4A	phosphodiesterase 4 A	enzyme	1.34
ENSG00000065154	OAT	ornithine aminotransferase	enzyme	1.33
ENSG00000095794	CREM	cAMP responsive element modulator	transcription regulator	1.32
ENSG00000108861	DUSP3	dual specificity phosphatase 3	phosphatase	1.31
ENSG00000108828	VAT1	vesicle amine transport 1	transporter	1.31
ENSG00000104687	GSR	glutathione-disulfide reductase	enzyme	1.22
ENSG00000100221	JOSD1	Josephin domain containing 1	peptidase	1.20
ENSG00000091592	NLRP1	NLR family pyrin domain containing 1	peptidase	−1.24
ENSG00000043143	JADE2	jade family PHD finger 2	enzyme	−1.28
ENSG00000087301	TXNDC16	thioredoxin domain containing 16	enzyme	−1.31
ENSG00000099991	CABIN1	calcineurin binding protein 1	other	−1.32
ENSG00000026950	BTN3A1	butyrophilin subfamily 3 member A1	other	−1.36

Names, symbols, molecule types, and fold changes (contrast: Poor vs. Good SAH Outcomes) for the 20 mRNA predictors of 90 day SAH Outcome

**Fig. 1:**
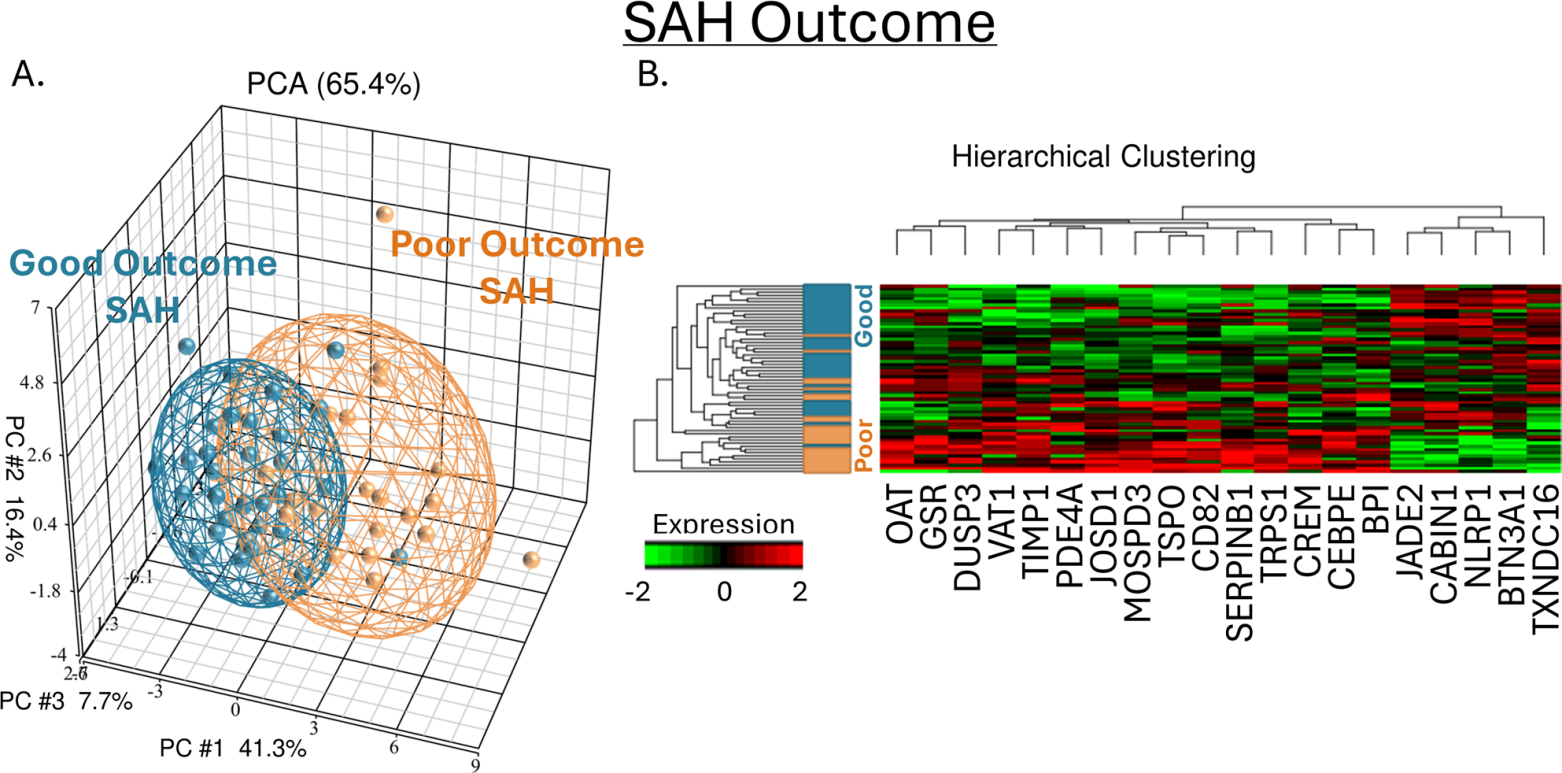
The 20 predictors of SAH Outcome separate Good and Poor SAH Outcome on Principal Components Analysis (PCA; **A**) and Hierarchical Clustering (HC; **B**). On the left PCA, each sphere represents a subject with Poor (orange) or Good (blue) SAH Outcome. Ellipsoids represent 2 standard deviations around the centroid of each group. On the right, both genes (columns) and subjects (rows; also displaying Poor as orange and Good as blue) underwent hierarchical clustering which showed distinct gene expression responses based on SAH outcome

Principal component analysis (PCA, Fig. 1A) and hierarchical clustering (Fig. 1B) of the 20 predictor genes from the derivation cohort showed good separation of Good SAH Outcome and Poor SAH Outcome patients. Separation on PCA was not driven by sex (SFig. 2), age (SFig. 3), hypercholesterolemia (SFig. 4), vasospasm (SFig. 5), clinical site (SFig. 6), diabetes (SFig. 7), race (SFig. 8), hypertension (SFig. 9), or delayed cerebral ischemia (DCI; SFig. 10).

### Best model performance on validation cohort

**Fig. 2:**
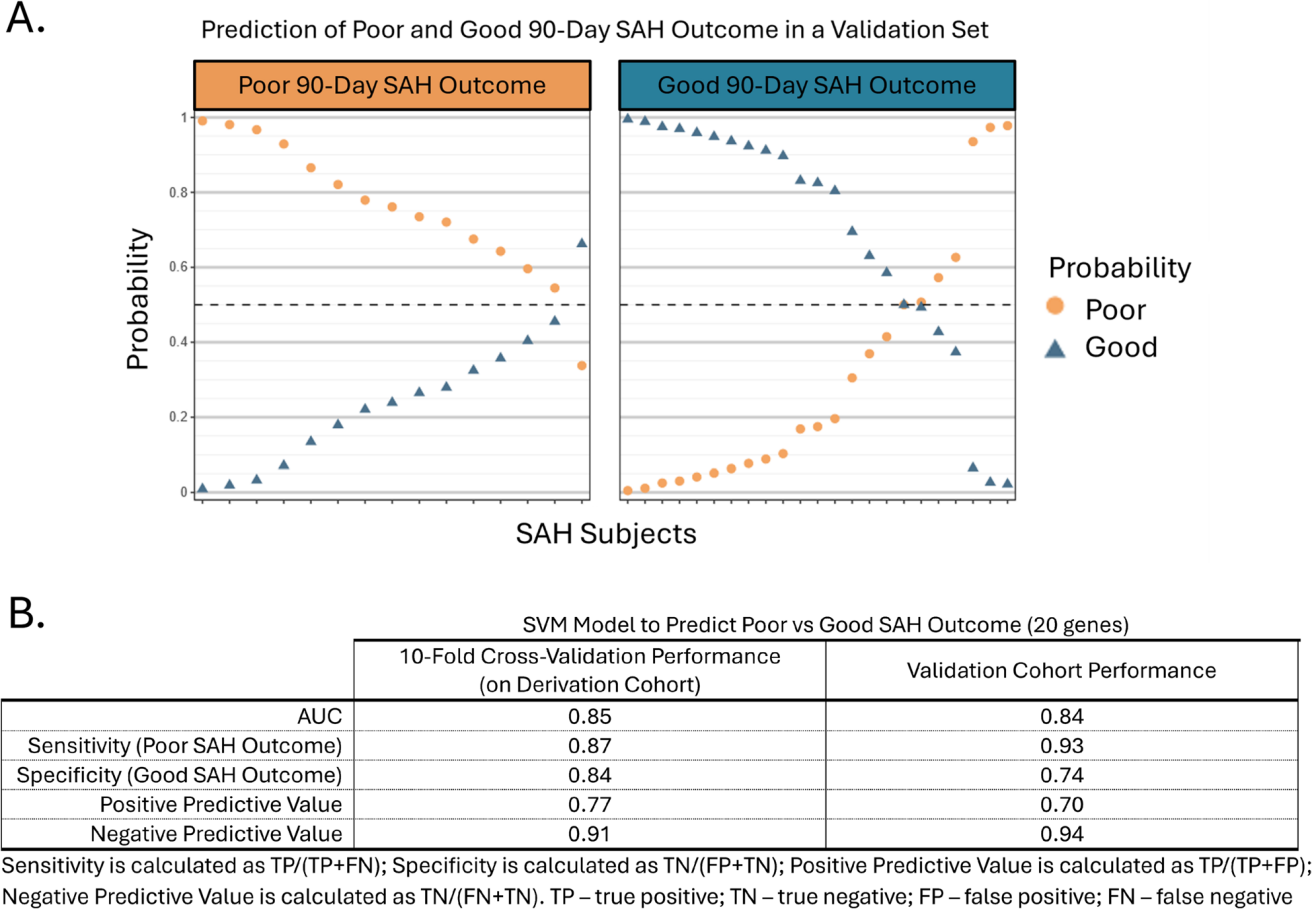
Validation cohort prediction probabilities of SAH outcome (**A**) and model performance in derivation and validation cohorts (**B**). In (**A**), the left panel displays the true Poor SAH Outcome subjects while the right panel displays the true Good SAH Outcome subjects. For each subject (x axis), the predicted probability (y axis) of having Poor (orange circle) versus Good (blue triangle) outcome at 90 days is displayed. A cutoff of probability = 0.5 is represented by a dashed black line

The selected model was then deployed on the Validation cohort (15 Poor, 23 Good SAH Outcome patients) to better estimate generalizability. This model correctly predicted 14 of 15 Poor SAH Outcome and 17 of 23 Good SAH Outcome patients. It attained an AUC of 0.84, a sensitivity of 93%, and a specificity of 74% (Fig. 2). When the validation cohort was broken down into aneurysm and non-aneurysm SAH participants, the model still performed well on both groups (STable 1). A graphic of the Prediction Probabilities for the Validation Cohort is shown in Fig. 2A for Poor SAH Outcome subjects (Fig. 2A, orange) and Good SAH Outcome Subjects (Fig. 2A, blue).

### Biological relevance of the 20 predictor genes

**Fig. 3:**
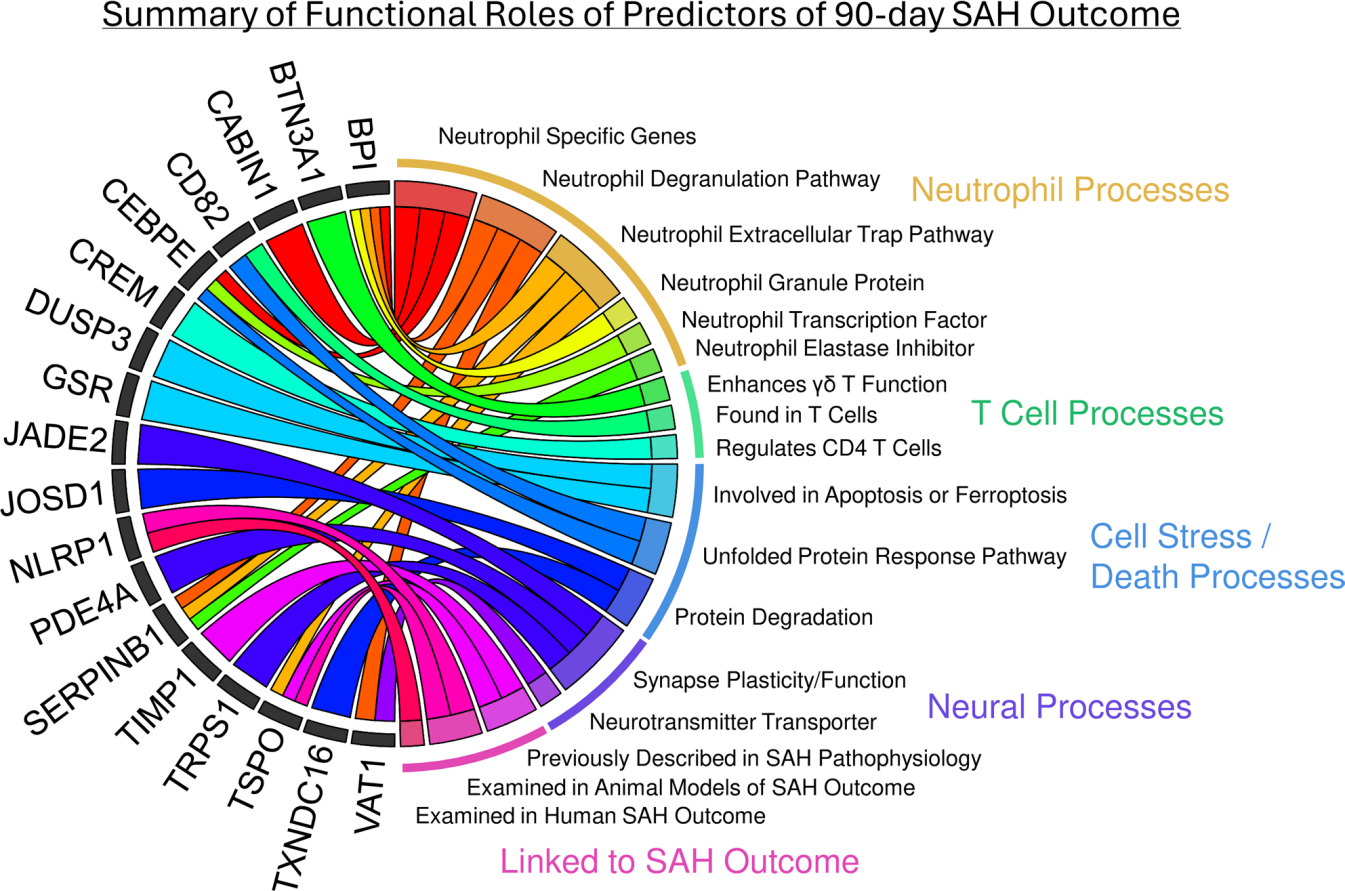
Summary of Functional Roles of Predictors of 90-day SAH Outcome. Some of the predictors with identified relevant functions are presented on the left of the diagram with chords connecting them to the functional roles on the right. Functional roles are grouped by their relevance to Neutrophil, T Cell, Cell Stress/Death, and Neural processes, as well as a group for genes previously linked to SAH pathophysiology or outcome. Functions from the literature are described in more detail in the Discussion section

The 20 predictor genes were significantly enriched (BH *p* < 0.05) in nine pathways (including Unfolded Protein Response, Neutrophil Degranulation, and Neutrophil Extracellular Trap Signaling; STable 2) as well as numerous Disease and Function terms in the Ingenuity Pathway Analysis (STable 3). The predictors were also significantly enriched in granulocyte- (mainly neutrophils) and erythroblast-specific genes (*p* < 0.05; STable 4). A summary of the predictors’ functional roles including pathways, functions, and relevant processes from the literature (described below in the discussion) is presented in Fig. 3.

## Discussion

This discovery study suggests the feasibility of using mRNA levels from a small group of genes expressed in peripheral blood in the early timeframe following SAH to predict 90-day functional outcomes as measured by mRS. We used machine learning to identify the best group of genes in a derivation cohort that predicted outcomes in a validation cohort with reasonable accuracy. We have previously shown that expression of a different group of genes in peripheral blood following SAH associates with those patients who develop vasospasm [22]. The current study also shows that the 20 outcome predictors were significantly enriched in granulocyte-specific genes (including neutrophils) and in nine pathways including the Unfolded Protein Response, Neutrophil Degranulation, and Neutrophil Extracellular Trap (NET) Signaling, as well as biofunctions including intracranial hemorrhage. Numerous pathways likely contribute to recovery following SAH, and neutrophil related pathways and genes may be of particular importance as potential targets that may influence stroke outcome trajectory. Importantly, biomarkers with relevance to the underlying biology of the injury of study can be more robust and generalizable across patients. Indeed, we observe many such links in our final marker panel for 90-day SAH outcome and these biological links are further described below.

Neutrophils, including their processes of degranulation and extracellular trap signaling, have been repeatedly linked to poor outcomes following SAH [23]. Persistent neutrophilia in peripheral blood of SAH patients has been associated with poor outcomes [24], and neutrophil-to-lymphocyte ratios, neutrophil-to-albumin ratios and increased numbers of neutrophils predict poor SAH outcomes [25]. A model using Neutrophil Extracellular Trap (NET) markers myeloperoxidase and elastase levels was predictive of cerebral vasospasm after SAH in humans [26]. In addition to their enrichment in neutrophil-related pathways, several of the 20 predictors of 90-day SAH outcome have been associated with neutrophil functions that could impact SAH outcome. One of these genes, bactericidal/permeability-increasing protein (BPI), is a human neutrophil granule protein that kills gram-negative bacteria by binding to lipopolysaccharide (LPS) and can reduce LPS-mediated cytokine production in monocytes and whole blood [27]. This functionality of BPI protects vascular endothelial cells from bacteria-mediated activation and injury [27]. A human recombinant BPI protein prevented burn-induced sequestration of neutrophils in tissues and tissue edema following burn injury [28]. CCAAT enhancer binding protein epsilon (CEBPE), a neutrophil transcription factor, modulates neutrophil development, proliferation, and differentiation, and is necessary for neutrophil granulopoiesis and anti-microbial and other neutrophil granule functions [29]. Serpin family B member 1 (SERPINB1) inhibits neutrophil elastase which is important in the formation of NETs, and generally suppresses inflammation and caspase activation in a variety of diseases and disease models [30]. These neutrophil related genes and functions may contribute to long term outcomes in SAH patients and as such may be potential treatment targets for improving outcome.

Several other predictors have been associated with various forms of T cells and could impact brain repair after SAH. Butyrophilin subfamily 3 member A1 (BTN3A1) enhances effector function of γδ T cells that are involved in tissue repair [31]. The tetraspanin CD82 is specific to oligodendrocytes in the CNS and is expressed during myelination [32] and may function to restrict precursor migration and aid in the transition from non-myelinating to myelinating oligodendrocytes [32]. CD82 contributes to T cell activation [32], is found in T lymphocytes and exosomes, and regulates S1PR_1_-mediated mobilization of hematopoietic stem and progenitor cells [33]. cAMP responsive element modulator (CREM), a transcription factor, regulates CD4 T cells via a number of interleukins, modulates adult hippocampal neurogenesis and memory consolidation and modulates astrogliogenesis [34–36]. Overall, these cellular immune responses to SAH could drive damage and neuronal repair, thus impacting long-term outcomes.

Additionally, some of the predictors are involved in inflammatory, cell death, and cellular stress responses that could drive outcomes after SAH. Dual specificity phosphatase 3 (DUSP3) regulates MAP kinases, is required to maintain epithelial tight junctions, decreases myocardial infarction induced apoptosis and inflammation, and modulates thrombosis, hemostasis and angiogenesis [37]. Glutathione-disulfide reductase (GSR), a potent antioxidant regulated by Nrf2, was shown to co-express with GPX4 in an animal ICH model, where increases in GSR and GPX4 were associated with decreases in ferroptosis [38]. The unfolded protein response, which has been shown to be neuroprotective in rodent SAH models, can be disrupted by SAH-associated ER stress [39]. The 20 predictors were enriched in the Unfolded Protein Response Pathway with CD82 and CEBPE overlapping. Josephin domain containing 1 (JOSD1) is a deubiquitinase that has been shown to prevent proteins from being marked for degradation in various cancers [40, 41]. TXNDC16 (thioredoxin domain containing 16), a member of the protein disulfide isomerase (PDI) family that promotes the formation and rearrangement of disulfide bonds, has been shown to co-immunoprecipitate with ERFAD (ER flavoprotein associated with degradation) and is hypothesized to play a role in protein recruitment for ER-associated protein degradation [42]. This upregulation of anti-degradation JOSD1 and downregulation of pro-degradation TXNDC16 indicates a decrease in the unfolded protein response with worse SAH outcomes, which is in line with the literature showing ER stress and unfolded protein responses being neuroprotective after SAH [39]. VAT1, an NADPH-dependent quinone oxidoreductase family member, may contribute to the regulation of mitochondrial fusion and phospholipid transport [43]. These responses also likely impact damage and repair mechanisms after SAH.

A number of predictors are also involved in neuronal and synaptic function and thus could contribute to neural repair post SAH. JADE2 (jade family PHD finger 2) promotes neurogenesis and shows increased expression with brain activity in mice [44]. JADE2 depletion in mouse neurons impaired synaptic plasticity, while its overexpression facilitated learning and memory through the hippocampus [44]. In this study, JADE2 was found downregulated in Poor Good SAH Outcome, while VAT1, PDE4A, and TRPS1 were found upregulated. Vesicle amine transport protein 1 (VAT1) is a protein in synaptic vesicle membranes that regulates the movement of monoamines like dopamine, epinephrine, and norepinephrine between synaptic vesicles and the cytosol in nerve terminals [43]. Phosphodiesterase 4 A (PDE4A) metabolizes the second messenger cyclic adenosine monophosphate (cAMP), modulates the CREB-BDNF pathway, and modulates synaptic plasticity and memory formation [45]. TRPS1 (transcriptional repressor GATA binding 1) is expressed in the developing brain and in adult brain astrocytes where it plays a role in maintaining synapses [46]. Drivers of neuronal and synaptic function such as these could impact long-term brain function in SAH patients.

Several of the predictors have previously been associated with SAH and its outcomes or have links to SAH recovery. The NLRP1 (NLR family pyrin domain containing 1) inflammasome facilitates the cleavage of caspase-1, leading to the maturation and subsequent secretion of pro-inflammatory factors interleukin IL-1β and IL-18. NLRP1 mediates inflammation following intracerebral hemorrhage [47], and NETs promote NLRP1-mediated neuronal pyroptosis. NLRP1 inhibition improves outcomes in Alzheimer’s disease, traumatic brain injury, ischemic stroke and SAH animal models [48]. High NLRP1 levels in CSF have previously been linked to poor 90-day outcomes following SAH in humans [49]. Ornithine aminotransferase (OAT), a mitochondrial enzyme, produces glutamate from ornithine [50] which could modulate SAH outcomes. TIMP1 is a tissue inhibitor of metalloproteinases (MMPs). MMPs are proteases that degrade various extracellular matrix (ECM) proteins, as well as many non-matrix substrates. TIMP1 directly suppresses inflammation by preserving ECM and decreasing inflammatory cell infiltration. TIMP1 plays a role in learning and memory and protects against blood brain barrier disruption and promotes ECM formation following SAH [51]. TIMP1 has previously been shown to decrease early and then increase at later times in peripheral blood following SAH in humans [51]. TSPO (translocator protein) is a mitochondrial protein expressed in activated microglia and has been used as an imaging target for neuroinflammation in a wide variety of neurological diseases and has been associated with molecular transport, decreasing oxidative stress and apoptosis, and sustaining energy metabolism [52]. TSPO increases in brain microglia after experimental SAH, and a ligand for TSPO administered after experimental SAH improved neurological function, increased anti-inflammatory molecules, and participated in signaling pathways that regulate microglial phenotype [53]. Molecules and mechanisms such as the ones discussed above may have potential as treatment targets given their association with SAH pathophysiology and outcomes.

In addition, we overlapped the 20 gene panel with gene lists from transcriptomic studies of other neurodegenerative diseases. We found significant overlap with gene lists associated with intracerebral hemorrhage (ICH) volume (SERPINB1, TSPO, and GSR) and absolute perihematomal edema (PHE; TSPO and GSR) volume in ICH patients [18]. As the hemorrhage itself is a major driver of damage after ICH and PHE is a major driver of secondary damage, both ICH and PHE volumes are highly associated with ICH outcomes [18]. Animal studies of ICH have shown a ligand for TSPO improved PHE and reduced brain injury [54]. Overlap between these ICH and PHE volume-associated genes and SAH outcome-associated genes could represent common mechanisms impacting long term outcomes in both hemorrhagic stroke types. In another study, BTN3A1 was also found differentially expressed in patients with ischemic stroke (significant overlap) and ICH versus controls [19]. This study discussed role of γδT cells especially in ICH [19]; this may also be a mechanism after SAH that impacts long term outcome. We also overlapped the predictors with a list of genes differentially expressed in Alzheimer’s Disease (AD) versus controls [20]. We found one gene (BPI) overlapped. BPI has been shown to be increased in AD compared to normal cognition and was a hub in a network analysis [55]. These overlapping genes may represent convergent mechanisms overlapping between these neurological/neurodegenerative diseases.

We describe a 20-gene panel that accurately predicts 90-day mRS outcomes following human SAH based on the early peripheral blood transcriptome. Predictive models such as these could be useful in clinical settings for identifying the best treatment plan and in research settings for stratifying participants in future clinical trials. In addition, the 20 predictors are involved in many biological processes that are highly relevant to SAH outcome, without even considering the other 74 genes and pathways that are significantly different between Good and Poor SAH Outcome patients. The many functions of the 20 predictors emphasize the complexity of the multiple molecules, functions, and pathways that must be involved in determining outcomes following SAH.

Limitations of the current study include the fact that it is a discovery study showing proof of principle that a peripheral blood gene profile can predict 90-day SAH outcomes. Much larger multi-site studies are needed to test the 20 predictors identified here for their generalizability to accurately predict 90-day SAH outcome. Lack of prior data prevented us from conducting a power analysis prior to this proof of principle study. However, we included a validation cohort to assess generalizability of the predictive model which showed strong performance of our model on a separate cohort. Other outcome measures should be also assessed in future trials including cognition and patient-reported quality of life. The predictors in the present study were derived from dichotomized outcome data, whereas future studies might also evaluate gene profiles that predict outcomes as an ordinal variable. Additionally, studies should examine the performance of the gene expression model in comparison to and when combined with clinical variables that are used to predict SAH outcomes. In our study, on a subset of patients where all data was available, the 20 gene profile alone performed superior to models using the Hunt Hess grade and age as well as models combining the 20 gene profile with Hunt Hess grade and age (data not shown).

## Supplementary Information

Supplementary Material 1.

Supplementary Material 2.

## Data Availability

The data underlying this article will be shared upon reasonable written request to the corresponding author.
