# Supplementary material for: Early peripheral blood gene expression predicts 90-day outcomes following subarachnoid hemorrhage
Source: J Neuroinflammation. 2025 Dec 7;22:286. doi: 10.1186/s12974-025-03630-0 (PMC12690849; doi:10.1186/s12974-025-03630-0)
Supplement: Supplementary file 2 — Supplementary Material 2. [file 12974_2025_3630_MOESM2_ESM.pdf]

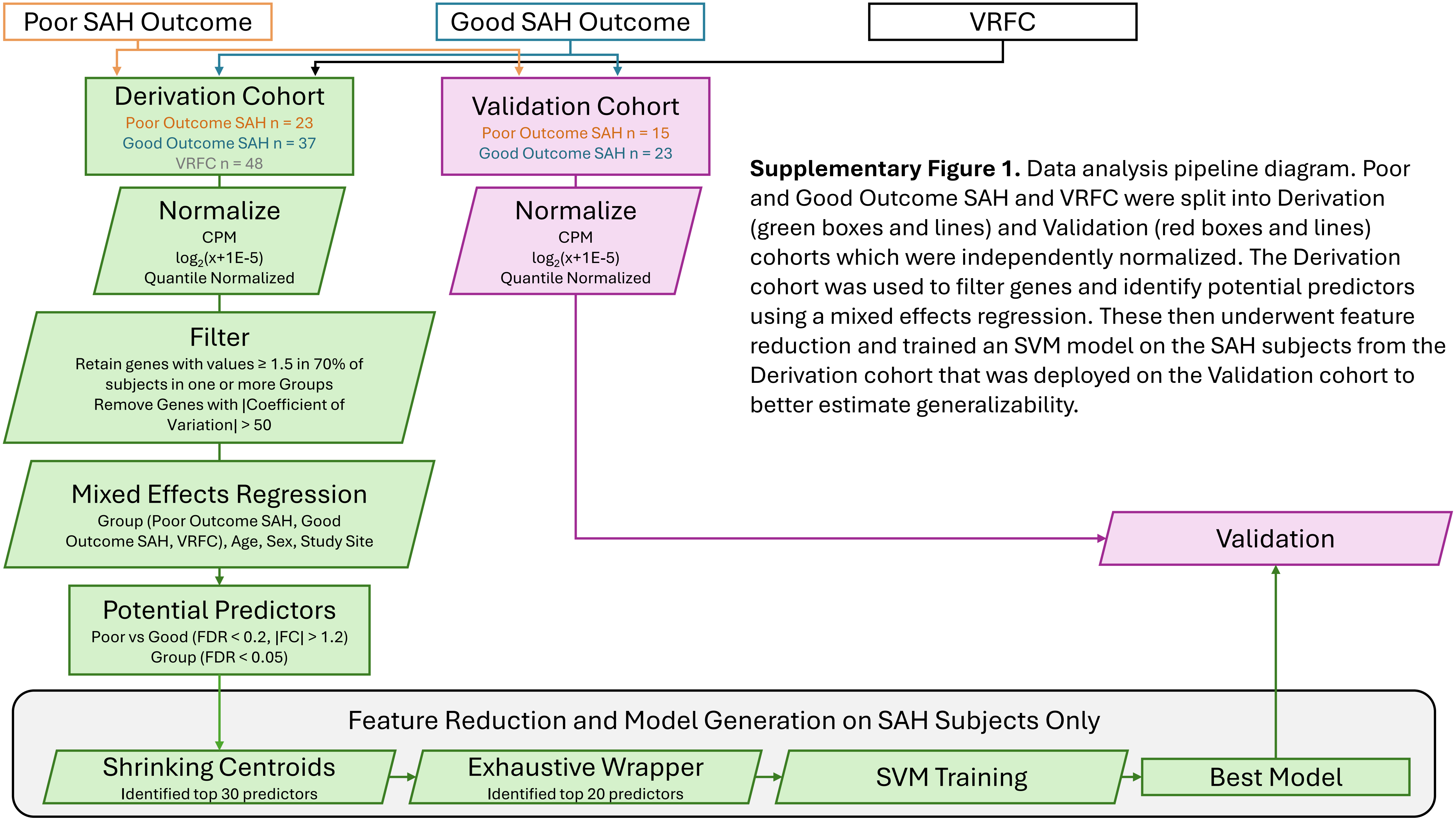

Supplementary Figure 1. Data analysis pipeline diagram. Poor and Good Outcome SAH and VRFC were split into Derivation (green boxes and lines) and Validation (red boxes and lines) cohorts which were independently normalized. The Derivation cohort was used to filter genes and identify potential predictors using a mixed effects regression. These then underwent feature reduction and trained an SVM model on the SAH subjects from the Derivation cohort that was deployed on the Validation cohort to better estimate generalizability.

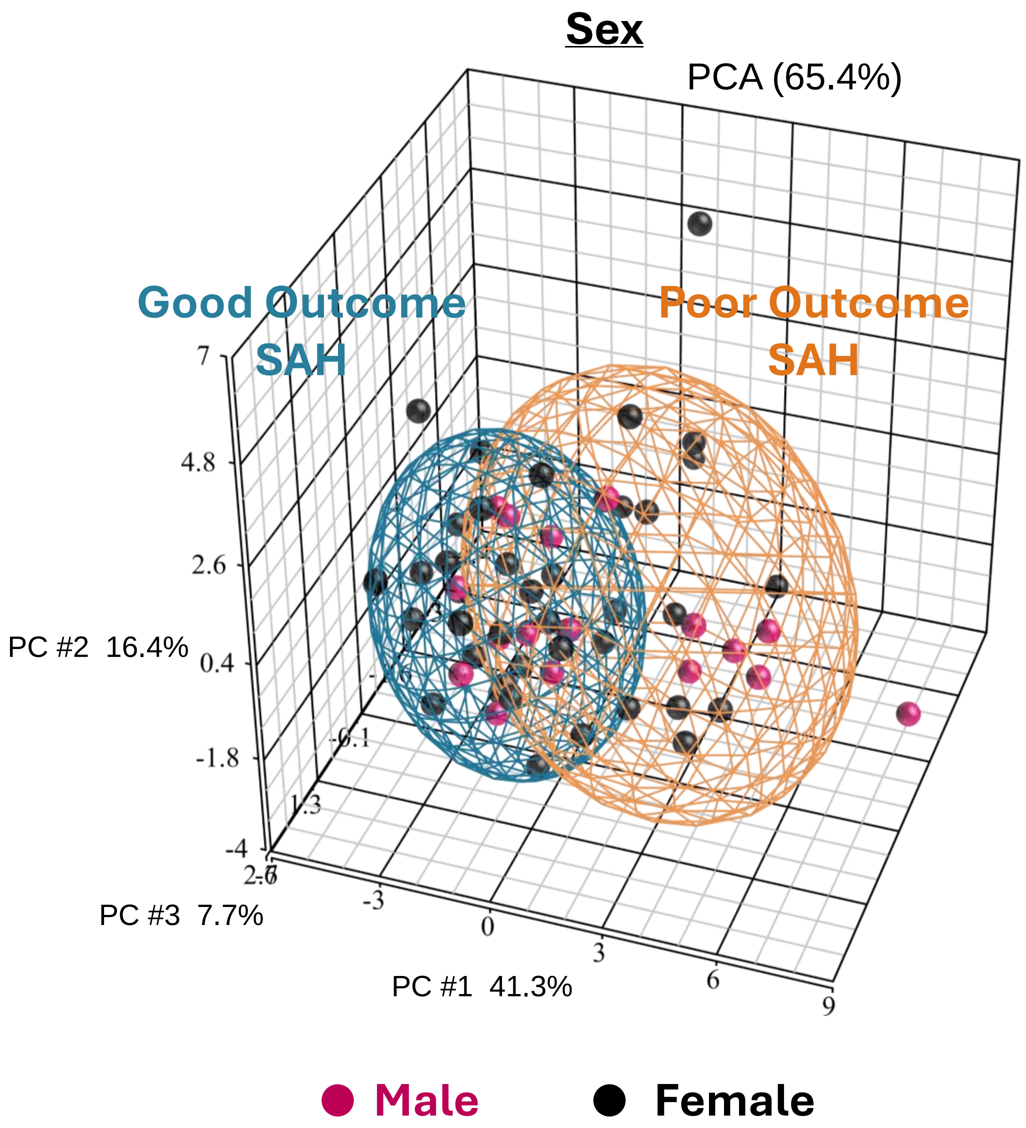

Supplementary Figure 2. PCA separation of the Derivation Cohort Poor and Good Outcome SAH Subjects was not driven by sex. Each sphere represents a subject colored by sex, and the ellipsoids represent 2 standard deviations from the centroid of the Good Outcome SAH and Poor Outcome SAH subjects.

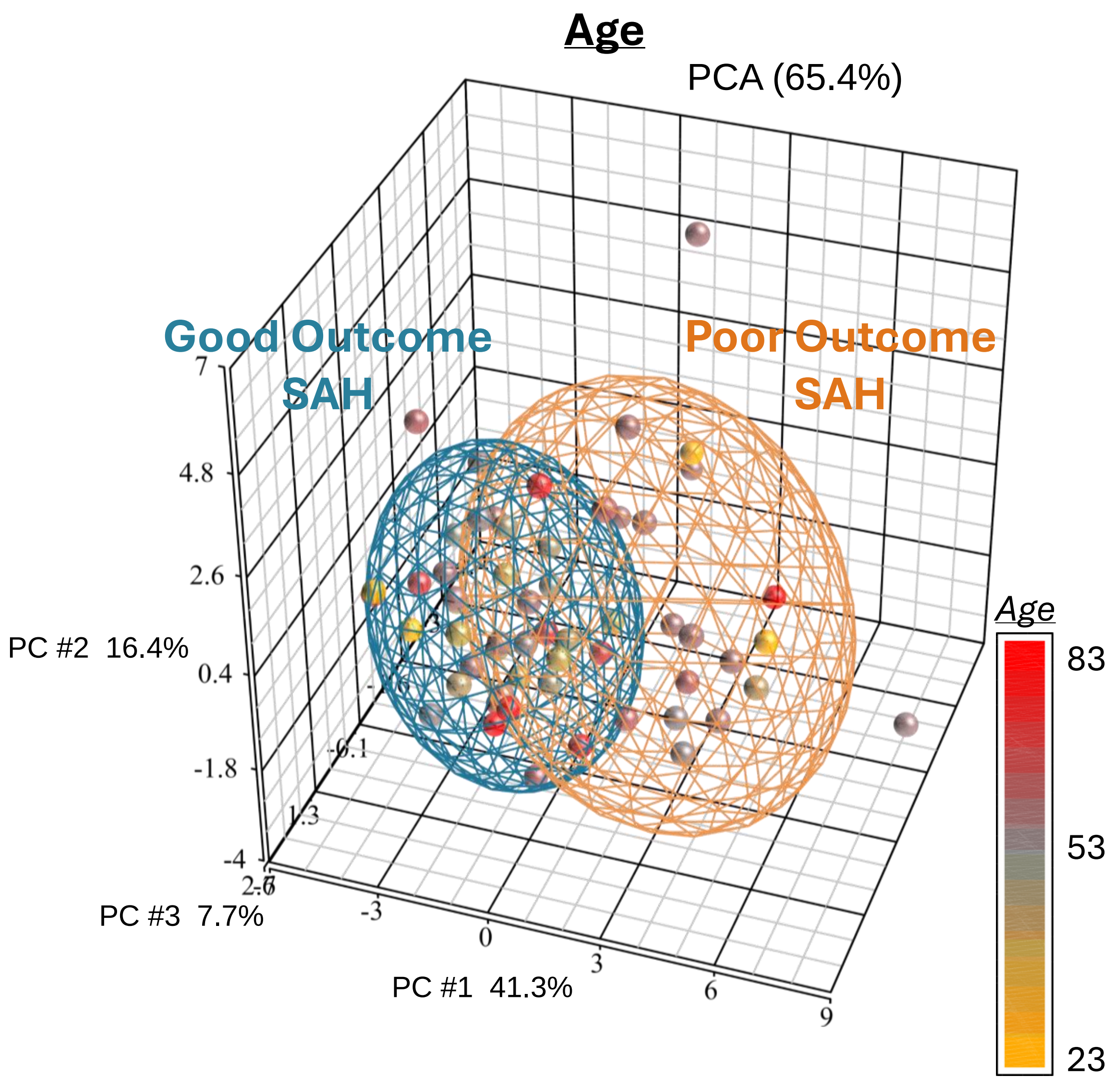

Supplementary Figure 3. PCA separation of the Derivation Cohort Poor and Good Outcome SAH Subjects was not driven by age. Each sphere represents a subject colored by age, and the ellipsoids represent 2 standard deviations from the centroid of the Good Outcome SAH and Poor Outcome SAH subjects.

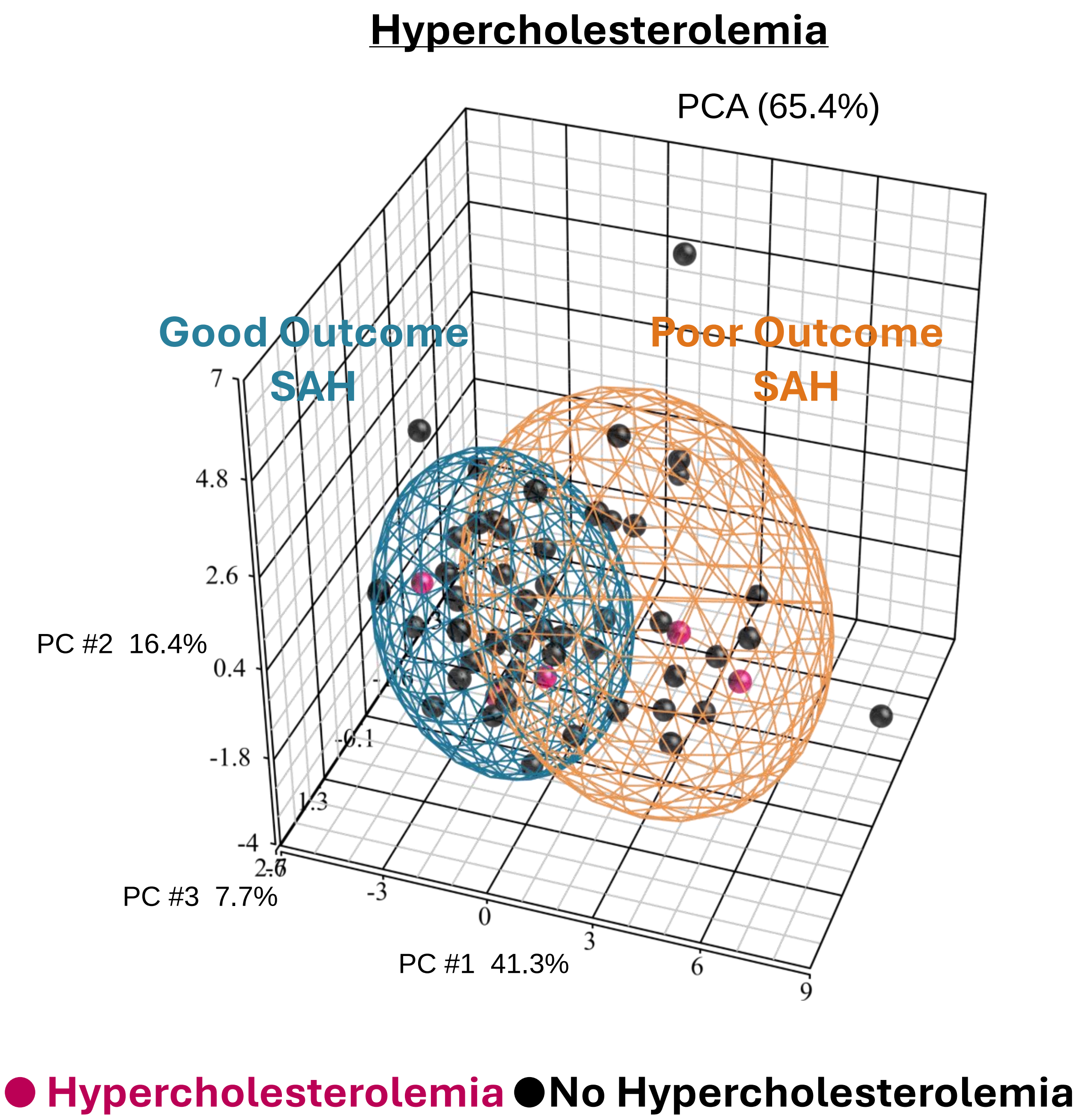

Supplementary Figure 4. PCA separation of the Derivation Cohort Poor and Good Outcome SAH Subjects was not driven by hypercholesterolemia. Each sphere represents a subject colored by hypercholesterolemia, and the ellipsoids represent 2 standard deviations from the centroid of the Good Outcome SAH and Poor Outcome SAH subjects.

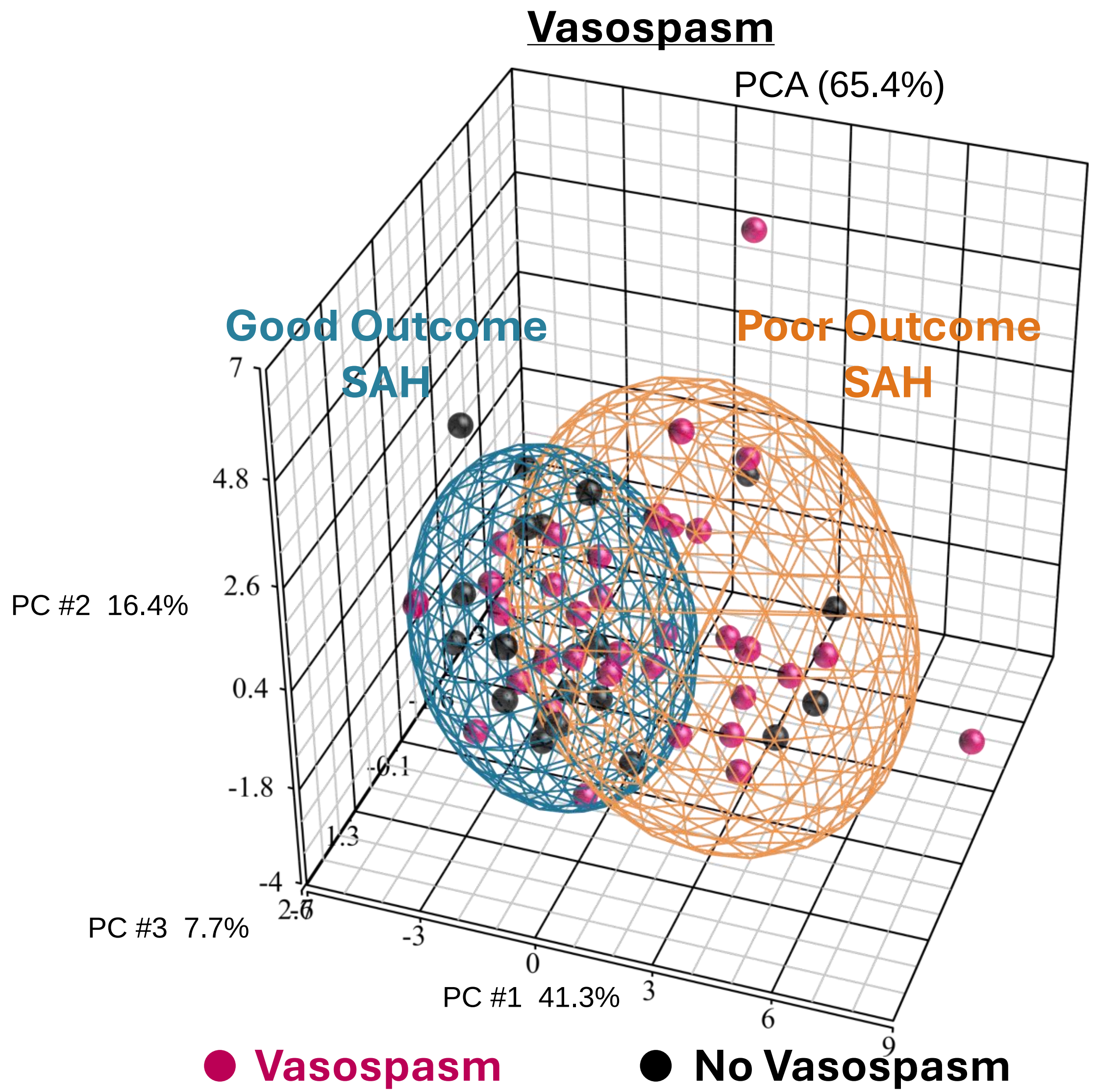

Supplementary Figure 5. PCA separation of the Derivation Cohort Poor and Good Outcome SAH Subjects was not driven by vasospasm. Each sphere represents a subject colored by vasospasm, and the ellipsoids represent 2 standard deviations from the centroid of the Good Outcome SAH and Poor Outcome SAH subjects.

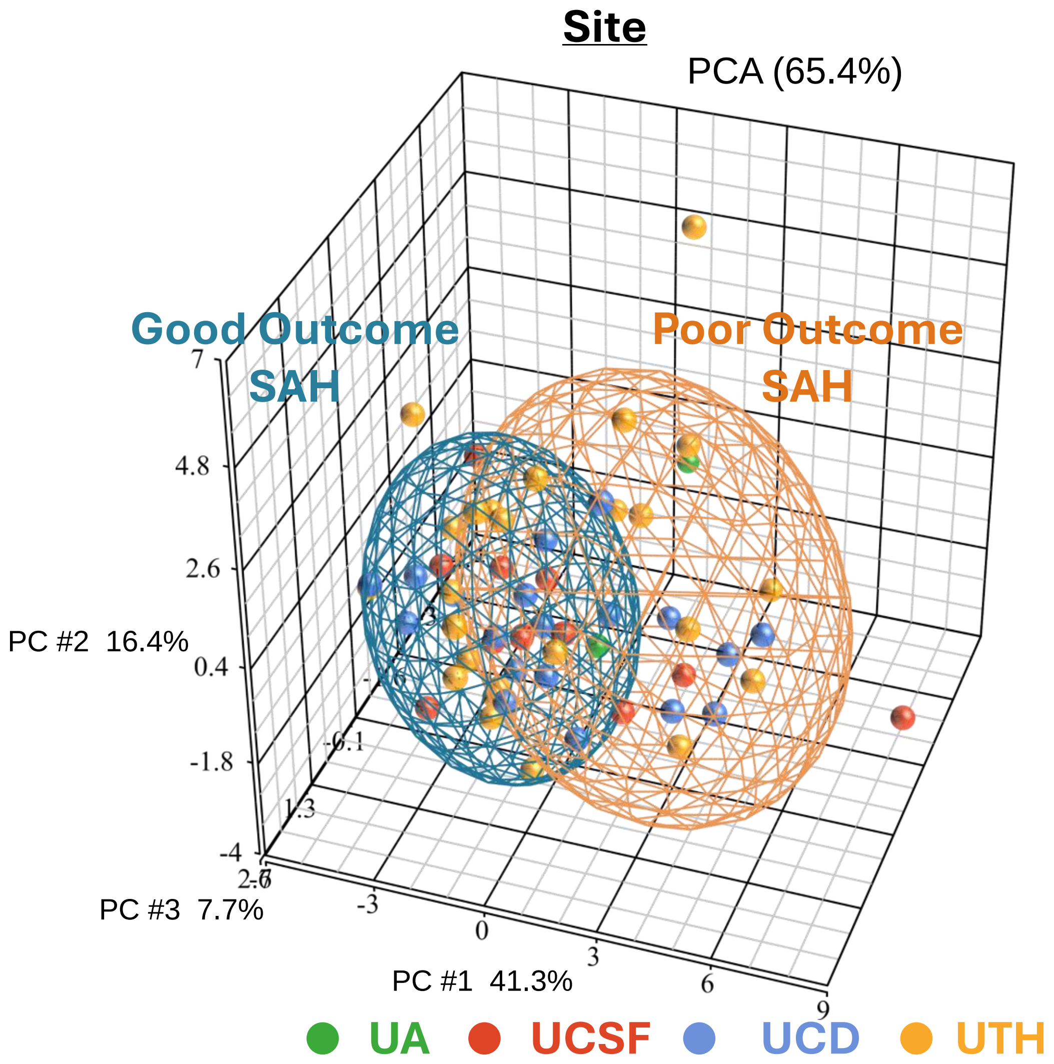

Supplementary Figure 6. PCA separation of the Derivation Cohort Poor and Good Outcome SAH Subjects was not driven by study site. Each sphere represents a subject colored by site, and the ellipsoids represent 2 standard deviations from the centroid of the Good Outcome SAH and Poor Outcome SAH subjects.

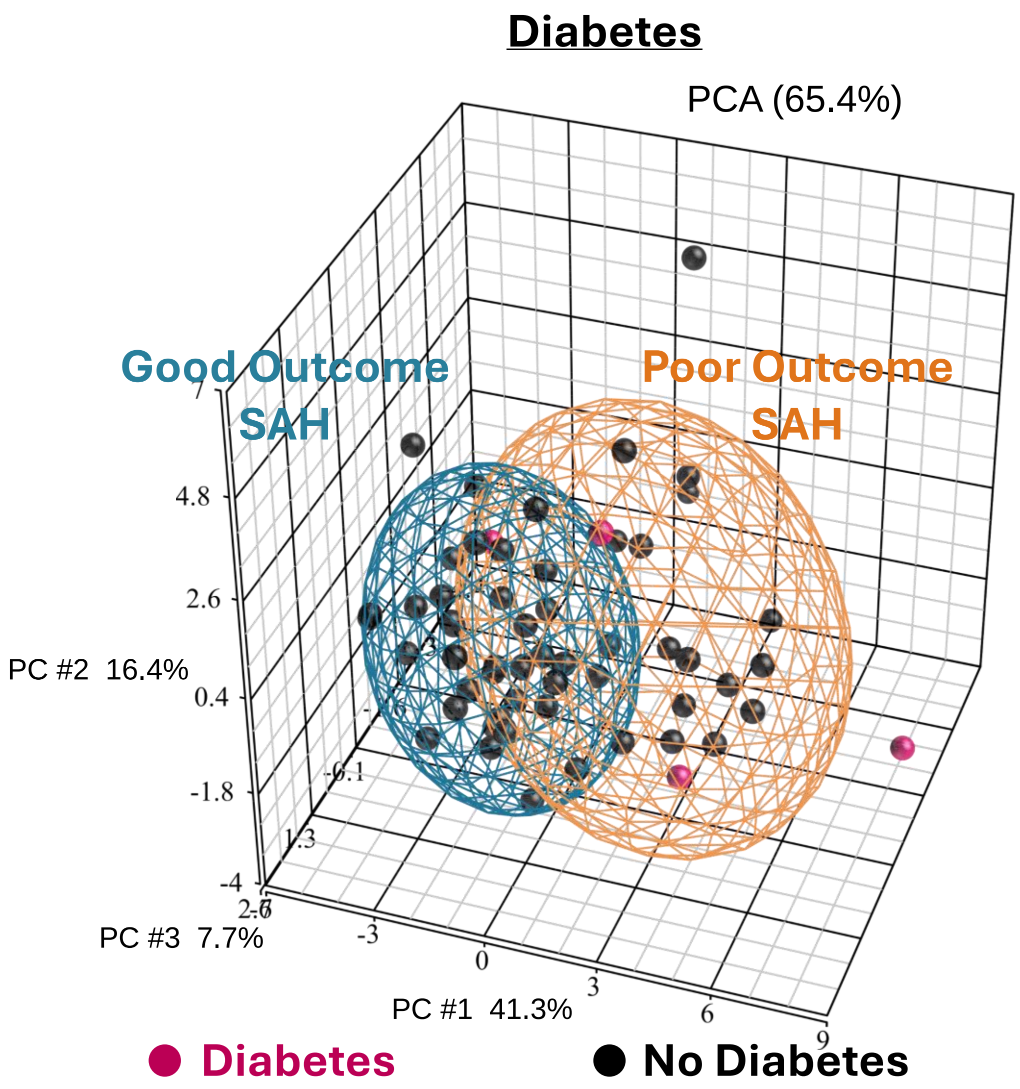

Supplementary Figure 7. PCA separation of the Derivation Cohort Poor and Good Outcome SAH Subjects was not driven by diabetes. Each sphere represents a subject colored by diabetes, and the ellipsoids represent 2 standard deviations from the centroid of the Good Outcome SAH and Poor Outcome SAH subjects.

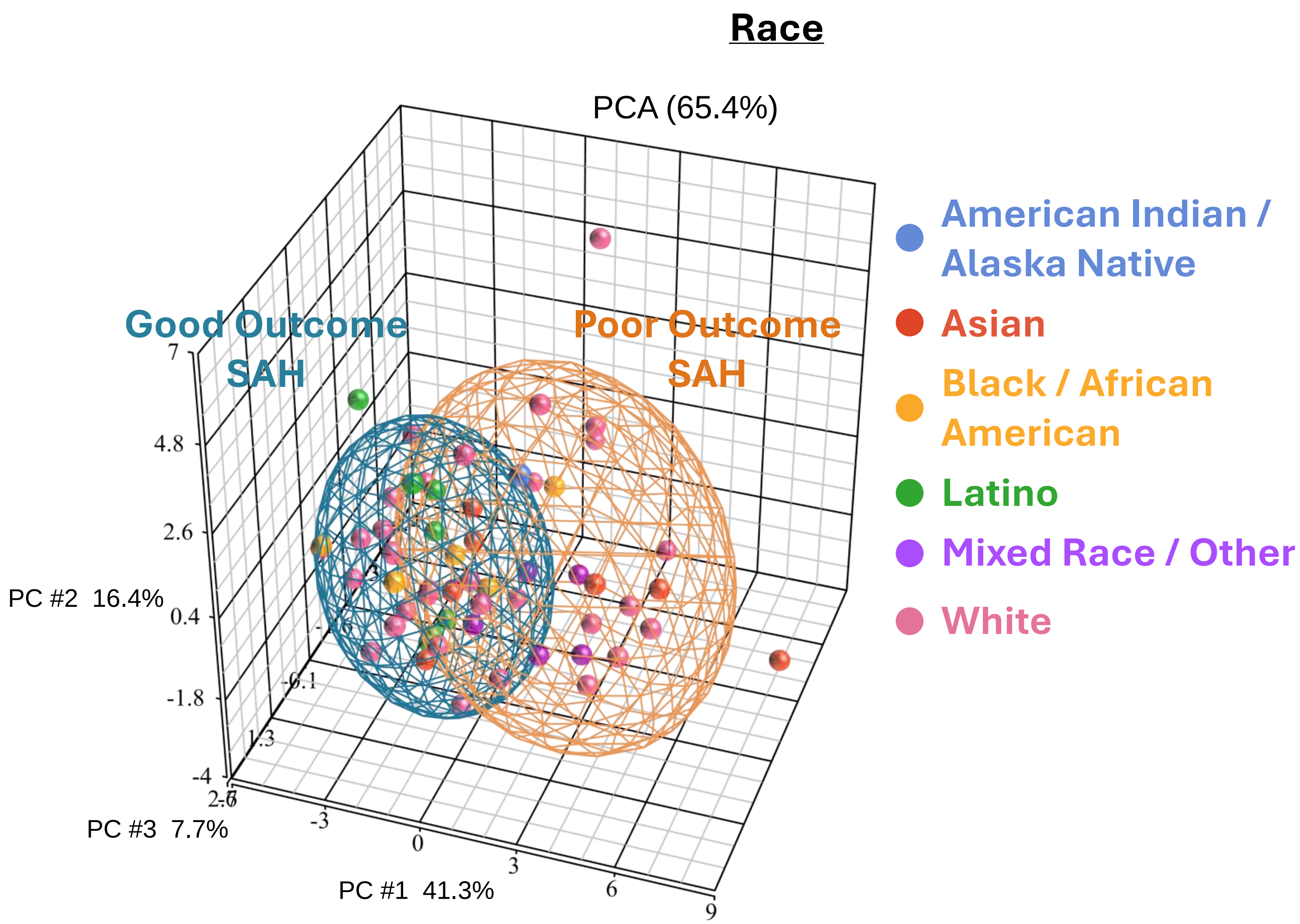

Supplementary Figure 8. PCA separation of the Derivation Cohort Poor and Good Outcome SAH Subjects was not driven by race. Each sphere represents a subject colored by race, and the ellipsoids represent 2 standard deviations from the centroid of the Good Outcome SAH and Poor Outcome SAH subjects.

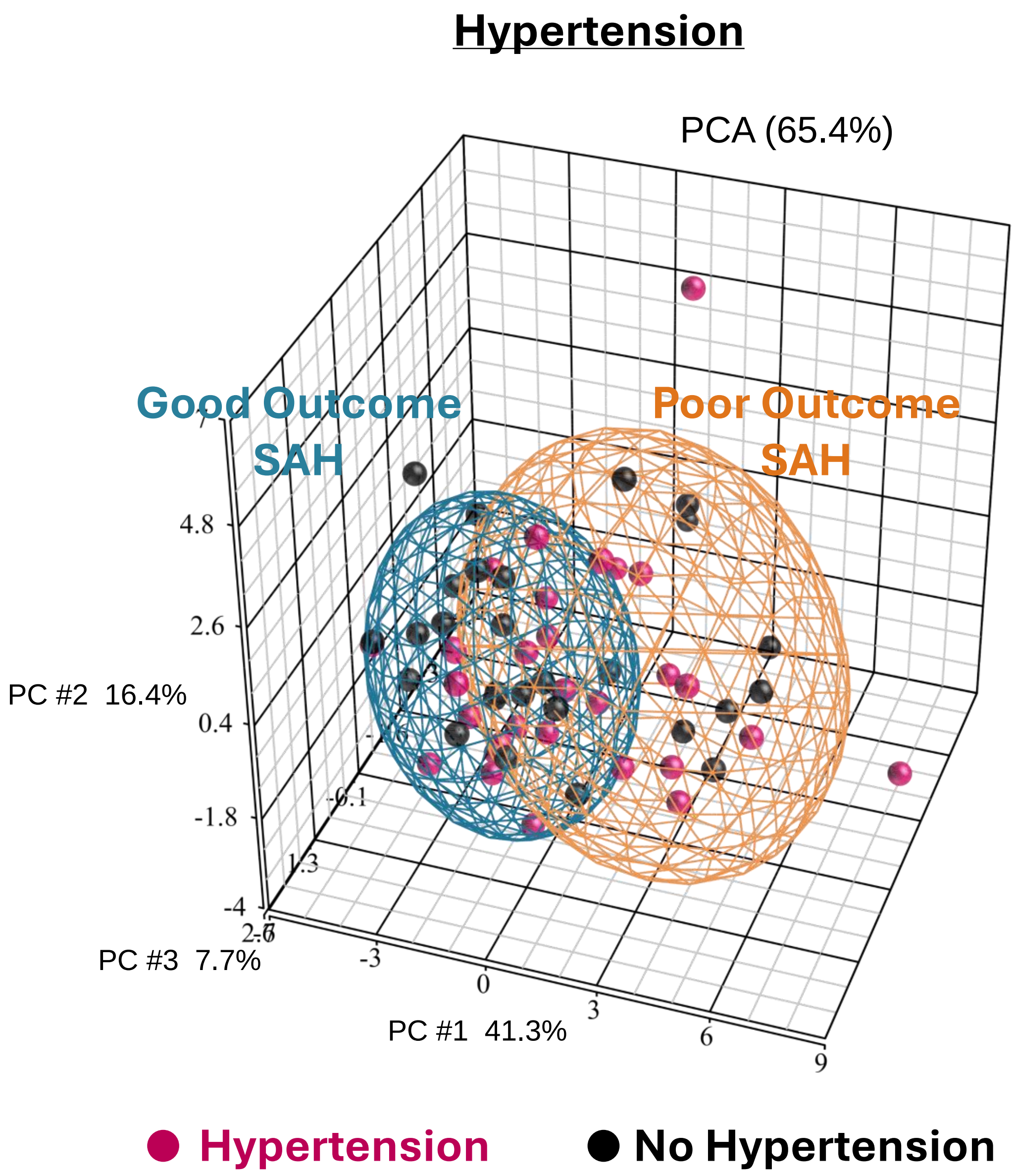

Supplementary Figure 9. PCA separation of the Derivation Cohort Poor and Good Outcome SAH Subjects was not driven by hypertension. Each sphere represents a subject colored by hypertension, and the ellipsoids represent 2 standard deviations from the centroid of the Good Outcome SAH and Poor Outcome SAH subjects.

Delayed Cerebral Ischemia (DCI)

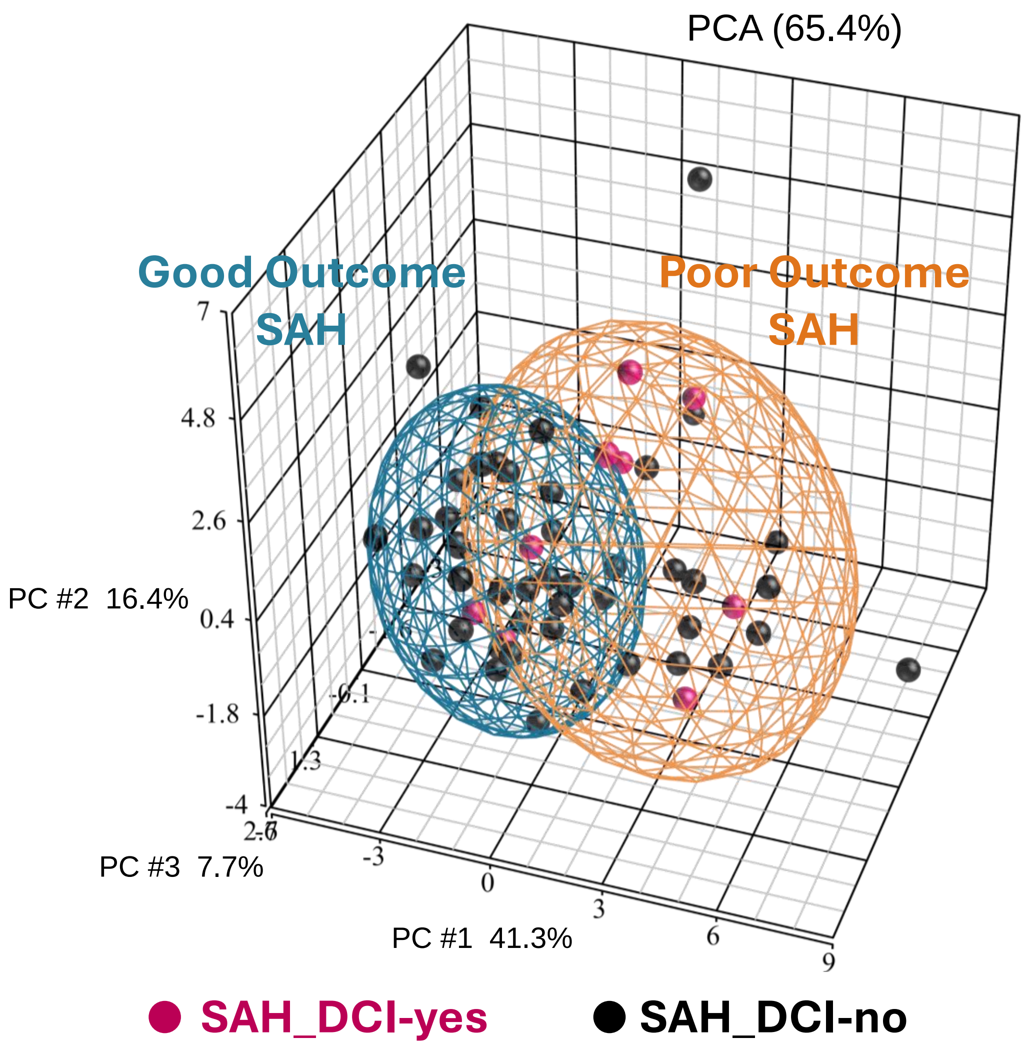

Supplementary Figure 10. PCA separation of the Derivation Cohort Poor and Good Outcome SAH Subjects was not driven by DCI. Each sphere represents a subject colored by DCI, and the ellipsoids represent 2 standard deviations from the centroid of the Good Outcome SAH and Poor Outcome SAH subjects.
